# The Association Between Religiosity and Lifelong Cancer Incidence in an Israeli Male Cohort: A Competing Risk Survival Analysis

**DOI:** 10.3390/epidemiologia7020038

**Published:** 2026-03-03

**Authors:** Lipaz Varkel, Uri Goldbourt, Yariv Gerber

**Affiliations:** Department of Epidemiology and Preventive Medicine, School of Public Health, Gray Faculty of Medical and Health Sciences, Tel Aviv University, P.O. Box 39040, Ramat Aviv, Tel Aviv 6997801, Israel; lipaz.varkel@gmail.com (L.V.);

**Keywords:** religiosity, cancer incidence, survival analysis, competing risks, cohort study

## Abstract

Background: While religious involvement has been linked to better health outcomes, its specific association with cancer incidence remains uncertain. The potential for confounding by lifestyle factors, such as physical activity, body weight, and smoking, complicates the interpretation of this relationship, necessitating further research in large, well-defined cohorts. This study aims to investigate the association between religiosity and cancer incidence in a large Israeli cohort while controlling for a comprehensive set of confounders and the competing risk of mortality. Methods: We conducted a retrospective analysis of 8746 male city-hall employees from the Israeli Ischemic Heart Disease (IIHD) cohort, enrolled in 1963. Cancer and mortality follow-up lasted through 2019. Religiosity was self-reported at baseline and categorized as secular, traditional, or religious. We employed a cause-specific Cox proportional hazards model with age as the time scale to analyze the risk of cancer incidence, treating death as a competing risk. The model was adjusted for a comprehensive set of baseline confounders, including socioeconomic status, smoking, physical activity, body mass index, systolic blood pressure, cholesterol, and diabetes. Results: During the follow-up period, cancer was diagnosed in 2692 participants. We observed a significant inverse association between religiosity and cancer incidence. Compared to secular participants, the religious group had a significantly lower risk of cancer (multivariable-adjusted hazard ratio [HR] = 0.80, 95% CI: 0.73–0.87; *p* < 0.001); the traditional group had a nonsignificantly lower risk (HR = 0.91, 95% CI: 0.82–1.02; *p* = 0.10). This association was specific to cancer incidence, as religiosity was not significantly associated with the competing risk of mortality. Conclusions: In this cohort study, a higher level of religiosity was associated with a significantly lower risk of lifelong cancer incidence, independent of a wide range of lifestyle, social, and clinical factors. These findings suggest that psychosocial and biobehavioral pathways associated with a religious lifestyle may play a protective role in cancer etiology.

## 1. Introduction

The association between religiosity and cancer incidence has been explored in several epidemiological and psychosocial studies, with mixed findings [1,2,3,4,5]. Evidence from studies of Christian religious communities suggests that lower cancer incidence observed in these populations is primarily attributable to healthier lifestyle behaviors (e.g., lower rates of smoking and alcohol use, healthier diets) that are often promoted within these communities, rather than religiosity itself exerting a direct protective effect [6,7,8,9]. When analyses adjust for lifestyle factors, the association between religiosity and reduced cancer risk is no longer significant, indicating that lifestyle mediates the observed relationship [1].

Systematic reviews and meta-analyses indicate that while religiosity and spirituality are associated with improved physical health and well-being among cancer patients, there is no consistent evidence that religiosity reduces cancer incidence or cancer-specific mortality. Studies examining the prognostic role of religiosity on cancer progression or survival have yielded inconsistent results [1,2,3].

Religiosity may influence cancer screening behaviors, with some evidence suggesting that frequent attendance at religious services is associated with increased participation in cancer screening, which could indirectly affect cancer incidence through earlier detection [9,10]. However, this effect varies by religious group and is not universally observed. Psychosocial pathways, such as enhanced social support within religious communities, better coping mechanisms for life stressors, and a greater sense of purpose, may also play a role in the link between religiosity and cancer incidence [11,12,13,14].

Israel provides a unique societal context for studying this association. The Israeli Ischemic Heart Disease (IIHD) cohort, established in the 1960s, offers a valuable opportunity to examine this relationship over a long-term follow-up period of more than 50 years, with detailed baseline data on a wide range of potential confounders [15,16,17,18,19,20].

This study aims to investigate the association between self-defined religiosity and the long-term risk of cancer incidence in a cohort of Israeli males. Using a competing risk survival model, we sought to determine if such an association exists after comprehensively adjusting for socioeconomic, lifestyle, and clinical factors and accounting for death as a competing risk.

## 2. Materials and Methods

### 2.1. Study Population

This study utilizes data from the IIHD cohort, a prospective study established in 1963. The cohort, which includes an updated outcome dataset through 2019, has a follow-up period of 54 years. The original cohort included 10,059 male Israeli civil servants aged 40 and over. Participants were recruited from municipalities in Tel Aviv, Haifa, and Jerusalem. Upon enrollment in 1963, all participants underwent a comprehensive baseline examination, including a medical history, physical measurements, and detailed questionnaires on demographics, lifestyle, and health behaviors. For this analysis, we included participants with complete data on religiosity and all model covariates, resulting in a final analytical cohort of 8746 men. All participants provided oral consent to participate in the study upon recruitment, after receiving explanations about the study objectives and the planned long-term follow-up. This occurred prior to the establishment of Ethical Review Boards in Israel. In addition, the Tel Aviv University Ethical Review Board approved the linkage between the IIHD database, the Israel Population Registry, and the Israel National Cancer Registry to monitor participants’ vital status and cancer incidence [15,16,17,18,19,20].

### 2.2. Variables and Measurements

Exposure: The primary exposure was religiosity, assessed at study enrollment through a detailed, self-administered questionnaire. Participants were asked to rate their level of religious observance on a scale from 1 (most religious/orthodox) to 5 (least religious/secular). They also reported the type of educational system they attended (religious, secular, or combined). From these two variables, a three-category composite variable was constructed to classify participants as “secular,” “traditional,” or “religious.” For all subsequent analyses, the secular group was designated as the reference category (Table 1) [18].

Outcome: The primary outcome was incident cancer of any type. The secondary outcome, treated as a competing risk, was death from any cause with no prior cancer. Event data were obtained through linkage with the Israel National Cancer Registry (INCR), which is the national repository for cancer data in Israel. Linkage was performed using unique national identification numbers assigned to all Israeli citizens. The INCR has been operational since 1961, and reporting has been mandatory by law since 1982, ensuring comprehensive national coverage, with an estimated 94% completeness of ascertainment for solid tumors [21].

A comprehensive set of potential confounding variables was selected based on established risk factors for cancer in the scientific literature. All covariate data originated from self-administered questionnaires, laboratory tests, and anthropometric measures completed during the study’s recruitment phase in 1963.

Smoking Status: Given that tobacco use is a leading cause of cancer, smoking behavior was assessed [22,23,24,25,26]. Participants self-reported their smoking habits and were categorized into mutually exclusive groups: “Never smoked” (reference), “Ex-smoker,” “1–10 cigarettes/day,” “11–20 cigarettes/day,” “21+ cigarettes/day,” and a separate category for pipe or cigar users.

Socioeconomic Status: To account for the complex influence of social standing on health outcomes, a composite socioeconomic status (SES) index was constructed [27,28,29,30,31]. This index, ranging from 1 (lowest) to 5 (highest), was based on a combination of participants’ years of schooling, possession of a high school or university diploma, and their salary grade, which was categorized into professional, administrative, teaching, technician, or laborer roles. This multi-component approach, previously established in this cohort [32], offers a more nuanced measure of SES than any single indicator.

Physical Activity: work-related and leisure-time physical activities were assessed separately [33,34,35,36,37]. Work-related activity was classified based on occupational demands into five categories: “Mainly sitting at a desk” (reference), “Mainly sitting at the wheel of a car,” “Mainly standing,” “Mainly walking,” and “Mainly physical work.”

Leisure-time activity was self-reported and categorized into four levels of intensity and frequency: “Almost none” (reference), “Sporadic” (e.g., occasional walks), “Light” (e.g., regular walking or cycling for at least 30 min), and “Energetic” (e.g., sports or vigorous activity multiple times per week).

Clinical and Anthropometric Measures: A panel of objective clinical measurements was performed at the baseline examination during study recruitment:

Blood pressure was measured. For interpretability in regression models, systolic blood pressure (SBP) was treated as a continuous variable and rescaled to 10 mmHg units [38,39,40]. Total cholesterol was determined from nonfasting serum samples [41,42]. Body mass index (BMI) was calculated as weight in kilograms divided by the square of height in meters (kg/m^2^). Both height and weight were measured [43,44]. Diabetes was ascertained as a dichotomous variable (yes/no) based on either a self-reported history of physician-diagnosed diabetes for which the participant was receiving treatment, or a diagnosis confirmed via an oral glucose tolerance test (OGTT) conducted at the baseline examination [45,46,47].

### 2.3. Statistical Analysis

To visualize the unadjusted probability of a cancer diagnosis over time, we plotted the cumulative incidence function (CIF) across religiosity groups. This method was chosen over the standard Kaplan–Meier approach to properly account for death as a competing risk. Unlike the Kaplan–Meier method, which censors competing events and can overestimate the probability of the primary outcome, the CIF provides a more realistic estimate of incidence by calculating the probability of cancer occurring in the presence of competing events that can remove an individual from the at-risk population [48].

We used a cause-specific Cox proportional hazards model to estimate the hazard ratios (HRs) and 95% confidence intervals (CIs) for the association between religiosity and cancer incidence. This approach is suitable for handling competing risks, where an individual may experience a different event (in this case, death) that precludes the primary outcome from occurring.

Age was used as the time scale in the model. The starting point was defined as the age at study entry, and the end point was the age at which an event occurred or censoring took place. This method appropriately adjusts for the strong effect of age on cancer incidence. The final multivariable model was adjusted for the following confounders: smoking, SES, physical activity, and clinical measures, namely SBP, total cholesterol, and BMI. All data preparation and statistical analyses were performed using R version 4.5.1 with the survival and dplyr packages.

The proportional hazard (PH) assumption for the final cause-specific Cox model was formally tested for each covariate and globally with the cox.zph() function. The assumption was found to hold for our primary exposure variable, indicating that religiosity’s effect was reasonably constant over the follow-up period. While violations of the PH assumption were noted for several covariates (smoking status, work activity, and SES), the validity of the HRs for our primary exposure was not compromised. The HRs for covariates with violated assumptions were interpreted as the average effect over the entire follow-up period.

## 3. Results

The final analytical cohort included 8746 men. During the follow-up period, 8629 events occurred: 2692 incident cancers and 5937 deaths among participants with no prior cancer.

The baseline characteristics of the study population are presented by religiosity groups, distributed as 3753 secular, 1395 traditional, and 3598 religious (Table 2). There were some notable differences among the groups. Religious participants exhibited a higher proportion of never smokers (38.0%) compared with secular (27.7%) and traditional (30.5%) participants, and a lower proportion of 21+ cigs/day smokers (9.8% vs. 16.0% and 14.6%, respectively). In terms of work activity, a smaller percentage of religious participants reported mainly sitting at a desk (36.2%) compared with secular (49.7%) and traditional (44.3%), with a higher proportion engaged in physical work (17.1% vs. 8.7% and 10.3%). Regarding SES, a substantial difference was observed. The religious group had a considerably higher proportion in SES Level 1 (lowest SES) at 35.2%, compared with 14.2% in the secular group and 18.6% in the traditional group. Conversely, the secular group had higher proportions in the top two SES levels (15.1% in Level 4 and 14.3% in Level 5) compared with the religious group (6.5% and 6.4%, respectively) and the traditional group (10.0% and 7.2%, respectively).

Mean age at study entry, SBP, and BMI were similar among the groups, with small standardized mean differences. The proportion of participants with diabetes was slightly higher in the religious and traditional groups compared to the secular group (7.4% and 7.0% vs. 5.3%, respectively). Cholesterol levels were slightly lower in the religious group (203.1 mg/dL) compared to the secular (212.5 mg/dL) and traditional (206.6 mg/dL) groups. Leisure activity patterns also showed some differences, with the religious group having the highest proportion reporting almost no leisure activity (63.2%).

### 3.1. Association Between Religiosity and Cancer Incidence

**Figure 1:**
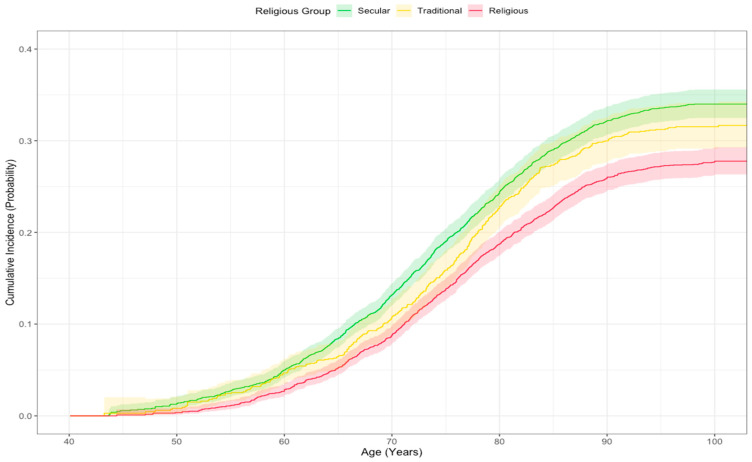
Cumulative incidence function (CIF) for cancer diagnosis by religious group.

**Figure 2:**
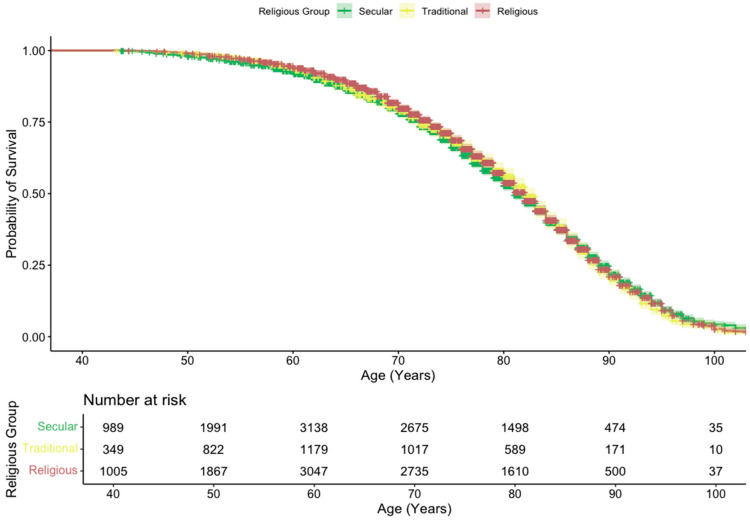
Kaplan–Meier survival curve by religious group.

The unadjusted cancer incidence rates per 100,000 person-years were 1269 (CI: 1201–1340) for secular participants, 1127 (CI: 1026–1238) for traditional participants, and 997 (CI: 937–1061) for religious participants. A CIF was used to visualize the unadjusted probability of cancer diagnosis by age, accounting for death as a competing risk. The resulting CIF curves showed a clear separation between the groups (Figure 1). The religious group demonstrated a consistently lower cumulative incidence of cancer, while the secular group showed the highest. In contrast, the survival curves for the competing event of death were nearly identical across all three religious groups, indicating no significant difference in mortality (Figure 2).

In a Cox PH model, after covariate adjustment, we found a statistically significant inverse association between religiosity and the risk of cancer incidence (Table 3). Compared to the secular group, participants who identified as religious had a 20% lower risk of being diagnosed with cancer (HR = 0.80; 95% CI: 0.73–0.87; *p* < 0.001). The association for the traditional group was not statistically significant (HR = 0.91; 95% CI: 0.82–1.02; *p* = 0.10).

### 3.2. Association with Mortality

In the analysis of the competing event, religiosity was not significantly associated with the risk of death. This suggests that the protective association observed was specific to cancer etiology rather than overall mortality. In contrast, several conventional risk factors, including smoking, diabetes, and SES, were strongly and significantly associated with death (Table 4).

### 3.3. Heterogeneity of Cancer Types

To formally test whether the association of religiosity with cancer risk was consistent across different cancer types, a meta-analytic approach was employed. The HRs for the religious versus secular groups were extracted from separate models for the most frequent cancer types in the cohort: prostate, lung, colon, and bladder cancer (Table 5). The formal test for heterogeneity was not statistically significant (Cochran’s Q = 3.17, *p* = 0.37), with a very low proportion of variation attributable to heterogeneity (I^2^ = 5.3%). Analyses of less frequent cancer types were not performed due to the limited number of events, which precluded reliable statistical estimation. This suggests that the protective association of religiosity did not differ significantly across the major cancer types studied.

## 4. Discussion

In this large, long-term cohort study of Israeli men, we found a significant inverse association between a high level of religiosity and the incidence of cancer. This relationship remained robust after adjusting for a comprehensive set of socioeconomic, lifestyle, and clinical confounders. The association also persisted after properly accounting for death as a competing risk.

Our findings are consistent with several large-scale studies that have identified an inverse association between religiosity and cancer risk. Studies of specific religious groups with prescribed healthy lifestyles have been particularly informative. For instance, the Adventist Health Studies have followed cohorts of Seventh-day Adventists for decades, consistently reporting lower rates of various cancers, including colorectal and lung cancer, compared to the general population. These benefits are largely attributed to their lifestyle, which often includes a vegetarian diet, regular physical activity, and abstinence from smoking and alcohol [4,6,7]. Similarly, a landmark study of California Mormons demonstrated significantly lower cancer mortality rates among active members who adhered to the “Word of Wisdom” health code, which prohibits tobacco and alcohol use [5,49,50,51].

A central debate in this field is the degree to which these associations are simply a product of confounding by healthier behaviors common in religious groups [7,8,51]. Indeed, some systematic reviews have concluded that after adjusting for healthy habits, the reduced risk for cancer is no longer observed, suggesting that lifestyle is the most important explanatory factor [1].

A notable feature of the present cohort is the distinct pattern of baseline characteristics observed across the three religious groups. The groups presented a complex mixture of protective and risk-associated factors at study entry. For instance, the religious group exhibited a healthier profile regarding certain key behaviors, with a significantly higher proportion of never-smokers (38.0%) and a lower average cholesterol level (mean of 203.1 mg/dL) compared with the secular group (mean of 212.5 mg/dL). However, this group also presented with factors typically associated with higher risk, including a much lower SES (35.2% in the lowest quintile vs. 14.2% of seculars) and lower levels of leisure-time physical activity. The secular group, conversely, had a higher socioeconomic profile but also a greater prevalence of heavy smoking. This complex interplay of lifestyle and sociodemographic differences underscores the critical importance of the multivariable adjustment performed in our analysis.

Our study makes a significant contribution by demonstrating that the inverse association between religiosity and cancer incidence persisted even after extensive adjustment for such lifestyle factors. This suggests that while health behaviors are undoubtedly important, they may not fully account for the observed protective association.

A potential limitation of our study is the possibility of detection bias, wherein differences in cancer screening behaviors between religious groups could influence incidence rates. While some international studies associate religious attendance with higher participation in preventive health services [9,10,52], research within Israel has indicated an inverse relationship. Studies have reported that more religious Israeli populations, particularly in ultra-Orthodox communities, exhibit lower adherence to cancer screening for malignancies such as colorectal cancer and breast cancer [53,54,55]. However, our site-specific analysis of colon cancer (Table 5) provides evidence against detection bias. In Israel, national guidelines recommend colorectal cancer screening for the average-risk population starting at age 50, making its detection rate particularly sensitive to screening behaviors. In our analysis, we found no significant difference in colon cancer incidence between the religious groups (HR for religious vs. secular = 0.98, 95% CI: 0.76–1.27). If underscreening were the primary driver of our overall findings, we would have expected to see a significant and artificially protective effect for a widely screened malignancy like colon cancer. The absence of such an association suggests that detection bias is unlikely to be the sole explanation for the lower risk observed.

The mechanisms linking religiosity to lower cancer risk are likely multifactorial. One of the most well-supported pathways is the provision of social support [12,56]. Religious communities often provide dense social networks that can buffer against stress and isolation, factors which have been linked to poorer health outcomes [57]. This support is a key mediator in the broader relationship between religious involvement and health.

Furthermore, religion can provide a framework of meaning, hope, and purpose that helps individuals manage life stressors more effectively [13]. This “stress-buffering” effect may translate into healthier physiological responses [58].

Observational and interventional studies have demonstrated that religious/spiritual activities—such as prayer, meditation, and communal worship—are associated with changes in neuroendocrine and immune parameters, including reductions in stress hormones (cortisol, epinephrine, norepinephrine) and pro-inflammatory cytokines, as well as improvements in immune function [14,59,60,61].

By mitigating stress, religious involvement may promote more favorable physiological profiles, such as lower levels of stress hormones (e.g., cortisol) or reduced inflammation, which in turn could influence cancer development [36,62,63,64].

The primary strengths of this study include its large cohort size, prospective design, and long-term follow-up, which allowed for the accumulation of a substantial number of incident cancer cases and provided the statistical power to detect meaningful associations. Furthermore, the availability of detailed baseline data on a wide range of covariates allowed for robust statistical adjustment, and the use of a competing risks model represents a significant methodological strength. 

Our study utilized a competing risks model to accurately estimate cancer incidence. This approach accounts for deaths from other causes, preventing overestimation and providing a more precise association between religiosity and cancer risk.

Our study has several limitations. First, the observational design precludes any inference of causality. Second, while our analysis adjusted for a comprehensive set of major confounders, the possibility of residual confounding from unmeasured factors still remains. For instance, our analysis did not include data on nutrition and alcohol consumption. These factors are strongly associated with both religiosity and cancer risk; therefore, residual confounding cannot be ruled out. However, it is worth noting that, per the literature, alcohol consumption in the Israeli population, particularly during the decades relevant to this cohort, is expected to be modest [65,66,67]. Factors specific to this population, such as adherence to a Kosher diet or the weekly day of rest (Sabbath), may also contribute to health outcomes and represent important avenues for future research. Additionally, information on participation in cancer screening during follow-up was not available. Furthermore, the study lacked validated measures of psychological distress, such as depression or anxiety; the absence of these variables prevents a full disentanglement of the effects of religiosity from general psychological well-being, as both are linked to health outcomes. Regarding marital status, a preliminary analysis indicated that the vast majority of participants (>93%) across all religiosity groups were married, rendering it unlikely to be a significant confounder. Third, the generalizability of our findings may be limited, as the cohort consisted exclusively of Israeli men. In this context, although approximately 13% of the original cohort were excluded due to missing baseline data, a comparison of key sociodemographic, clinical, and behavioral characteristics revealed that the excluded group was slightly older and had a somewhat less favorable cardiovascular risk profile.

Finally, religiosity and other lifestyle and health behaviors were assessed only at baseline, and this single-time-point measurement does not account for potential changes in participants’ religious beliefs or lifestyle over the nearly 50-year follow-up period.

Future research should aim to replicate these findings in more diverse populations, particularly among women and in various cultural contexts. Prospective studies that repeatedly measure religiosity would help to clarify how changes in belief and practice affect health outcomes. Most importantly, future investigations should incorporate the measurement of biological markers to directly test the hypothesized mediating pathways. Examining the interplay between religiosity, psychosocial factors, stress hormones, and markers of immune function and inflammation could provide crucial insights into the specific mechanisms underlying the religion–cancer link.

## 5. Conclusions

In conclusion, findings from this cohort of Jewish men in Israel provide evidence that a religious lifestyle is associated with a significantly lower risk of cancer incidence. This association is independent of many major confounders. The findings underscore the importance of considering psychosocial and biobehavioral pathways in cancer epidemiology, suggesting that the resources provided by a religious framework may have tangible, protective health benefits.

## Figures and Tables

**Table 1:** Classification matrix for the religious group variable based on religious practice as rows and religious education as columns. Secular (Green): Predominantly individuals with a secular education and lower levels of religious practice. Traditional (Yellow): Individuals with mixed educational backgrounds or moderate levels of religious practice. Religious (Red): Predominantly individuals with a religious education or the highest levels of religious practice.

	1. Religious Only	2. Secular & Religious	3. Secular Only	4. Did Not Learn or Category Was Unchecked
**1 (Most religious)**	Religious	Religious	Religious	Religious
**2**	Religious	Religious	Traditional	Religious
**3**	Religious	Traditional	Secular	Traditional
**4**	Traditional	Secular	Secular	Secular
**5 (Least religious)**	Secular	Secular	Secular	Secular

**Table 2:** Baseline characteristics of the study population by religiosity group.

Variable	Secular	Religious	Traditional	Standardized Mean Difference
*n*	3753	3598	1395	
Age at study entry (mean (SD))	49.64 (6.73)	50.02 (6.76)	48.90 (6.57)	0.11
Smoking (%)				0.22
Never smoked	1041 (27.7)	1369 (38.0)	426 (30.5)	
1–10 cigs/day	479 (12.8)	507 (14.1)	214 (15.3)	
11–20 cigs/day	760 (20.3)	647 (18.0)	291 (20.9)	
21+ cigs/day	600 (16.0)	351 (9.8)	203 (14.6)	
5+ cigar/pipe/day	59 (1.6)	27 (0.8)	8 (0.6)	
<5 cigar/pipe/day	50 (1.3)	33 (0.9)	21 (1.5)	
Ex-smoker	764 (20.4)	664 (18.5)	232 (16.6)	
Work activity (%)				0.24
Mainly sitting at desk	1867 (49.7)	1301 (36.2)	618 (44.3)	
Mainly sitting at wheel	181 (4.8)	110 (3.1)	69 (4.9)	
Mainly standing	484 (12.9)	516 (14.3)	179 (12.8)	
Mainly walking	864 (23.0)	1006 (28.0)	370 (26.5)	
Physical work (mainly)	325 (8.7)	616 (17.1)	144 (10.3)	
Does not work	28 (0.7)	47 (1.3)	13 (0.9)	
Other types of activity	4 (0.1)	2 (0.1)	2 (0.1)	
Systolic blood pressure (mean (SD))	137.61 (21.02)	138.58 (21.71)	137.42 (22.56)	0.03
Diabetes (%)	199 (5.3)	268 (7.4)	98 (7.0)	0.06
Cholesterol (mean (SD))	212.53 (38.25)	203.08 (38.99)	206.62 (36.58)	0.16
Leisure activity (%)				0.12
Almost none	2053 (54.7)	2275 (63.2)	797 (57.1)	
Sporadic	617 (16.4)	444 (12.3)	226 (16.2)	
Light	758 (20.2)	622 (17.3)	261 (18.7)	
Energetic	325 (8.7)	257 (7.1)	111 (8.0)	
Body mass index (mean (SD))	25.75 (3.06)	25.96 (3.48)	26.06 (3.20)	0.06
Socioeconomic status (%)				0.43
1	534 (14.2)	1266 (35.2)	260 (18.6)	
2	813 (21.7)	865 (24.0)	384 (27.5)	
3	1303 (34.7)	1000 (27.8)	510 (36.6)	
4	565 (15.1)	235 (6.5)	140 (10.0)	
5 (highest)	538 (14.3)	232 (6.4)	101 (7.2)	

**Table 3:** Hazard ratios for cancer incidence (primary outcome) from the multivariable cause-specific Cox model.

Variable	Hazard Ratio (HR)	95% Confidence Interval	*p*-Value
Religious Group			
Secular	1 (ref.)	1 (ref.)	
Traditional	0.91	0.82–1.02	0.1
Religious	0.80	0.73–0.87	<0.001
Smoking			
Never Smoked	1 (ref.)	1 (ref.)	
1–10 cigs/day	1.01	0.89–1.15	0.84
11–20 cigs/day	1.50	1.34–1.67	<0.001
21+ cigs/day	1.76	1.56–1.99	<0.001
5+ cigar/pipe/day	1.48	1.03–2.13	0.03
<5 cigar/pipe/day	1.08	0.79–1.48	0.64
Ex-smoker	1.05	0.93–1.17	0.49
Work Activity			
Sitting at Desk	1 (ref.)	1 (ref.)	
Sitting at Wheel	1.26	1.04–1.52	0.02
Mainly Standing	1.02	0.91–1.15	0.72
Mainly Walking	1.05	0.95–1.17	0.33
Physical Work	1.06	0.91–1.24	0.44
Does Not Work	0.96	0.65–1.43	0.84
Other	1.63	0.62–4.33	0.32
Socioeconomic Status			
SES Level 1	1 (ref.)	1 (ref.)	
SES Level 2	1.02	0.90–1.15	0.78
SES Level 3	1.02	0.90–1.16	0.73
SES Level 4	1.13	0.96–1.33	0.14
SES Level 5 (highest)	1.09	0.93–1.27	0.29
Leisure Activity			
Almost None	1 (ref.)	1 (ref.)	
Sporadic	1.05	0.94–1.17	0.42
Light	0.99	0.89–1.10	0.86
Energetic	0.99	0.86–1.14	0.85
Systolic Blood Pressure (per 10 mmHg increase)	1.02	1.00–1.04	0.03
Diabetes (yes vs. no)	0.87	0.71–1.07	0.19
Cholesterol (per 40 mg/dL increase)	0.99	0.95–1.03	0.54
Body Mass Index (per 1 kg/m^2^ increase)	1.00	0.99–1.02	0.67

**Table 4:** Hazard ratios for death incidence (competing risk) from the multivariable cause-specific Cox model.

Variable	Hazard Ratio (HR)	95% Confidence Interval	*p*-Value
Religious Group			
Secular	1 (ref.)	1 (ref.)	
Traditional	1.02	0.94–1.10	0.65
Religious	0.97	0.91–1.03	0.30
Smoking			
Never Smoked	1 (ref.)	1 (ref.)	
1–10 cigs/day	1.09	1.00–1.18	0.04
11–20 cigs/day	1.29	1.20–1.40	<0.001
21+ cigs/day	1.72	1.57–1.87	<0.001
5+ cigar/pipe/day	1.38	1.06–1.81	0.02
<5 cigar/pipe/day	0.92	0.71–1.18	0.50
Ex-smoker	1.10	1.03–1.18	0.01
Work Activity			
Sitting at Desk	1 (ref.)	1 (ref.)	
Sitting at Wheel	1.01	0.87–1.17	0.90
Mainly Standing	1.04	0.96–1.13	0.38
Mainly Walking	1.06	0.99–1.13	0.12
Physical Work	1.13	1.03–1.25	0.01
Does Not Work	1.30	1.00–1.69	0.05
Other	0.84	0.44–1.63	0.61
Socioeconomic Status			
SES Level 1	1 (ref.)	1 (ref.)	
SES Level 2	0.95	0.88–1.03	0.20
SES Level 3	0.90	0.83–0.97	0.01
SES Level 4	0.83	0.75–0.93	0.01
SES Level 5 (highest)	0.77	0.69–0.87	<0.001
Leisure Activity			
Almost None	1 (ref.)	1 (ref.)	
Sporadic	0.93	0.86–0.99	0.04
Light	0.94	0.88–1.01	0.09
Energetic	0.95	0.87–1.04	0.25
Systolic Blood Pressure (per 10 mmHg increase)	1.14	1.12–1.15	<0.001
Diabetes (yes vs. no)	1.89	1.70–2.10	<0.001
Cholesterol (per 40 mg/dL increase)	1.11	1.08–1.14	<0.001
Body Mass Index (per 1 kg/m^2^ increase)	1.01	1.00–1.02	0.03

**Table 5:** Hazard ratios for specific cancer types.

Cancer Type (Percentage in Cohort)	Traditional HR (95% CI)	Traditional *p*-Value	Religious HR (95% CI)	Religious *p*-Value
Prostate (16.1%)	0.92 (0.70–1.21)	0.56	0.76 (0.61–0.94)	0.01
Lung (11.4%)	0.81 (0.58–1.14)	0.22	0.72 (0.55–0.95)	0.02
Colon (10.9%)	0.91 (0.65–1.28)	0.59	0.98 (0.76–1.27)	0.88
Bladder (8.8%)	0.82 (0.56–1.20)	0.30	0.82 (0.61–1.10)	0.19

The models are adjusted for age, smoking, systolic blood pressure, diabetes, serum cholesterol, leisure-time physical activity, body mass index, and socioeconomic status. Hazard ratios are reported using the secular group as the reference. HR: Hazard Ratio.

## Data Availability

The data presented in this study are available upon request from the corresponding author. The data are not publicly available due to privacy restrictions regarding medical records and registry linkage.
